# Polycystic Echinococcosis in Pacas, Amazon Region, Peru

**DOI:** 10.3201/eid2103.141197

**Published:** 2015-03

**Authors:** Pedro Mayor, Laura E. Baquedano, Elisabeth Sanchez, Javier Aramburu, Luis A. Gomez-Puerta, Victor J. Mamani, Cesar M. Gavidia

**Affiliations:** Universitat Autònoma de Barcelona, Barcelona, Spain (P. Mayor);; Universidad Nacional Mayor de San Marcos, Lima, Peru (L.E. Baquedano, L.A. Gomez-Puerta, C.M. Gavidia);; Instituto Nacional de Salud, Iquitos, Peru (E. Sanchez, J. Aramburu);; Universidad Peruana Cayetano Heredia, Lima (V.J. Mamani)

**Keywords:** Polycystic echinococcosis, *Echinococcus vogeli*, *Cuniculus paca*, Amazonian Peruvian villages, prevalence, tapeworms, parasites

## Abstract

In the Peruvian Amazon, paca meat is consumed by humans. To determine human risk for polycystic echinococcosis, we examined wild pacas from 2 villages; 15 (11.7%) of 128 were infected with *Echinococcus vogeli* tapeworms. High *E. vogeli* prevalence among pacas indicates potential risk for humans living in *E. vogeli*–contaminated areas.

In the Peruvian Amazon, the presence of parasites associated with zoonotic diseases is affected by subsistence hunting, sale of bush meat for human consumption, and increased human contact with wild animals (*1*). One such zoonotic disease, polycystic echinococcosis, is caused by ingestion of food or water contaminated with *Echinococcus vogeli* tapeworm eggs and the subsequent development of larvae (cysts), mainly in the liver (*2*). Although this disease is seriously underreported, >200 cases in humans from 12 countries in Central and South America have been described (*2*–*4*); most confirmed cases were in Colombia and Brazil, and the first case in Peru was reported in 2004 (*2*,*5*).

Bush dogs (*Speothos venaticus*) are the most common definitive hosts for *E. vogeli* cestodes (adult cestode carriers; wild rodents, especially pacas (*Cuniculus paca*), may be the most common intermediate hosts (larvae or metacestode carriers) (*2*). In the Amazon region, pacas are among the most frequently hunted animals, and paca meat is highly commercialized in regional cities (K. Moya. Monitoreo de la comercialización de carne de monte en los mercados de Iquitos y estrategias para su conservación [undergraduate thesis]. Iquitos [Peru]: Universidad Nacional de la Amazonia Peruana; 2011). To verify the presence of *E. vogeli* cysts in wild pacas in 2 communities in the Peruvian Amazon (Nueva Esperanza and Diamante/7 de Julio), we examined paca viscera submitted by hunters.

## The Study

During June 2009–August 2013, as part of an ongoing participatory conservation program that involves hunters in community-based wildlife management, hunters collected thoracic and abdominal organs from pacas. The research protocol was approved by the Dirección General de Flora y Fauna Silvestre (0350-2012-AG-DGFFS-DGEFFS) from Peru. No pacas were killed solely for this study.

Viscera from 128 pacas were submitted for examination. Of the 120 pacas for which sex was known, 66 (55%) were female. A total of 120 viscera samples were obtained from pacas in Nueva Esperanza and 8 from pacas in Diamante/7 de Julio. 

Organs with cyst-like structures were analyzed at the San Marcos University School of Veterinary Medicine or the Peruvian National Institute of Health. Protoscolices collected from cysts were mounted in Berlese mounting medium. *E. vogeli* protoscolices were identified according to the shape and size of rostellar hooks (*6*). Tissue samples were fixed in 4% formalin before staining with hematoxylin and eosin for histopathologic examination. The Fisher exact test was used to analyze differences between communities, seasonality (wet and dry), and sex of pacas; significance was set at p<0.05.

Of the 128 pacas, polycystic echinococcosis was present in 15; overall prevalence was 11.7% (95% CI 6.7–18.6%). In Diamante/7 de Julio, prevalence was 25.0% (2/8; 95% CI 3.2%–65.1%), and in Nueva Esperanza, it was 10.8% (13/120; 95% CI 5.9%–17.8%; p = 0.24). The number of infected pacas differed between wet and dry seasons, but the difference was not significant (wet season = 13 infected pacas, dry season = 2; p = 0.14). No significant association between infection and sex was found (8 males and 7 females; p = 0.78).

**Figure 1:**
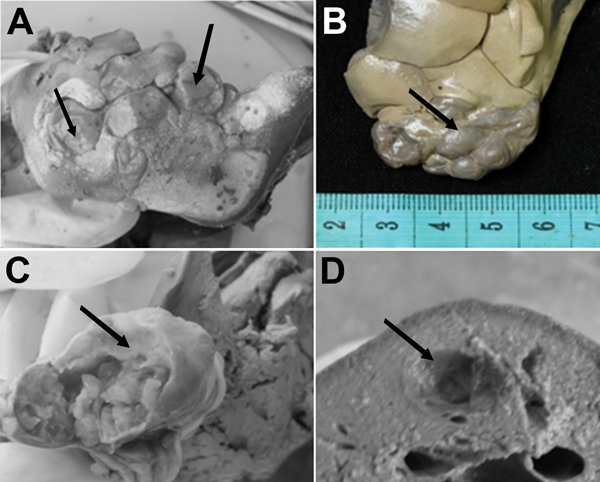
Multiple *Echinococcus vogeli* cysts (larval stage) in the liver of a wild paca. A, B) Vesicles exposed at the hepatic surface (arrows). C, D) Transected larvae in thick sections of liver showing internal structure of vesicles and characteristic distribution of brood capsules (arrows). Ruler in panel B indicates centimeters.

**Figure 2:**
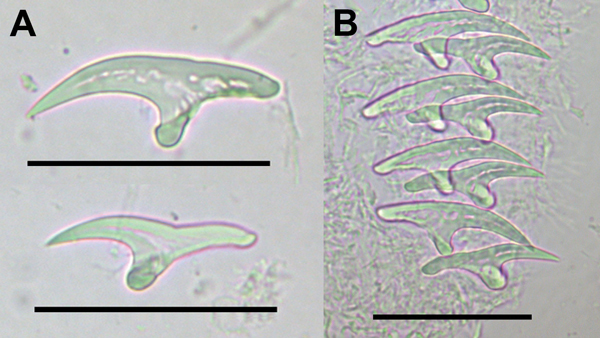
Large and small rostellar hooks from protoscolices of *Echinococcus vogeli*. A) Large (top) and small (bottom) hooks. B) Rows of rostellar hooks. Scale bars indicate 40 μm.

In 14 of the infected pacas, only liver cysts were found; in the other paca, cysts were found in liver and lungs (Figure 1). An average of 3.6 ± 8.2 liver cysts were found (1–52 cysts, 0.4–3.5 cm diameter). The only paca with cysts in the lungs had 8 such cysts, 0.2–1.0 cm diameter. The mean ± SD of the total length of large hooks was 41.9 ± 0.4 μm; the mean size of small hooks was 34.3 ± 0.9 μm. For large hooks, the handle length was 15.7 μm and the blade length was 27.6 μm. The number of hooks (large and small) ranged from 42 to 46 (Figure 2).

Histologic examination revealed metacestodal tissue (protoscolices, inner germinal layer, intermediate thick laminated layer, and outer adventitial layer) compressing hepatic parenchyma. The acellular laminated layer appeared convoluted, and eosinophils were seen after staining with hematoxylin and eosin. Intact laminated layers were lined on the inside by the germinal layer, which formed brood capsules and had free protoscolices. A thick and fibrous layer surrounded the liver cysts with an infiltration of macrophages, neutrophils, eosinophils, plasma cells, and lymphocytes.

## Conclusions

The high prevalence of polycystic echinococcosis in pacas in this study is consistent with the paca being a common intermediate host and the possibility that human cultural and dietary habits in the Amazon region might induce a parasite life cycle that involves domestic animals, particularly dogs. Most persons in the Amazon depend on subsistence hunting (*7*). They do not, however, consume the organs from hunted pacas; instead, they typically feed the viscera to their dogs, which might become definitive hosts. This feeding practice results in a high risk for introduction, development, and dissemination of *E. vogeli* cestodes in rural communities; humans might consequentially be infected through contact with feces from infected dogs. Other associated risk factors are poor hygienic conditions, unavailability of clean water, inadequate medical care, and insufficient knowledge about local diseases.

Among humans who receive medical attention, the polycystic echinococcosis fatality rate is 29%; causes of death are surgical accidents during cyst removal and direct parasite consequences (e.g., hepatic failure and its complications) (*2*,*3*). However, reported cases in humans might represent only the tip of the iceberg (*2*). Poor accessibility to rural jungle areas and lack of adequate public health infrastructures inhibit research among humans and wild animals (*8*), probably resulting in underestimation, inaccurate recording, or nondetection of cases (*2*,*3*). This neglected disease may cause chronic conditions that reduce productivity and income earning capacity.

High levels of bush meat commercialization in cities in the Amazon region of Peru (K. Moya, 2011) suggest direct contact among wildlife, humans, and domestic animals (*9*). The study area has not been affected much by humans; most contact between the local population and wildlife occurs during hunting activities. Outdoor activities, such as logging, oil extraction, or road construction, may cause human migration from urban areas to the rainforest, increasing risk for exposure to sylvatic pathogens (*10*) such as *E. vogeli* cestodes. Human movements introduce domestic animals, such as dogs, to forested ecosystems where they can encounter parasites or other infectious disease agents. Ecotourism also increases the likelihood of contact between tourists, wildlife, and potential pathogens (*11*).

This study was limited in that samples were neither random nor numerous, and the number of collected viscera differed between the 2 communities. Collection of paca viscera was part of the ongoing participatory conservation program; although hunters were trained to collect the viscera, sample availability was based on hunters’ willingness to participate. However, the infection rate we found might indicate an emerging public health problem in many countries; for instance, *E. vogeli* infections have been documented in a hunter from French Guyana (*12*), in an indigenous human population in the Amazon region of Venezuela (*13*), and in pacas and bush dogs in Argentina (*4*). The disease may also emerge in other countries, such as the Netherlands, where polycystic echinococcosis in a human was recently diagnosed (*14*).

Our study could be the beginning of more investigations involving wild and domestic animals and humans in the Amazon to determine the incidence and prevalence of polycystic echinococcosis in human populations and the potential role of domestic dogs as carriers of adult *E. vogeli* cestodes (*4*). The true prevalence and public health effects of this parasite must be estimated in communities in the Amazon region, and the search for *E. vogeli* infections should be expanded to other regions and countries.
